# Active pharmaceutical contaminants in drinking water: myth or fact?

**DOI:** 10.1007/s40199-024-00536-9

**Published:** 2024-09-18

**Authors:** Zvanaka Mazhandu, Tebogo Mashifana

**Affiliations:** Department of Chemical Engineering, University of Johannesburg, P.O. Box 17011, Doornfontein, 2088 South Africa

**Keywords:** Emerging contaminants, Pharmaceutical pollution, Clean water preservation, Water treatment methods

## Abstract

Global water availability has been affected by a variety of factors, including climate change, water pollution, urbanization, and population growth. These issues have been particularly acute in many parts of the world, where access to clean water remains a significant challenge. In this context, preserving existing water bodies is a critical priority. Numerous studies have demonstrated the inadequacy of conventional water treatment processes in removing active pharmaceutical ingredients (APIs) from the water. These pharmaceutical active compounds have been detected in treated wastewater, groundwater, and even drinking water sources. The presence of APIs in water resources poses a significant threat not only to aquatic organisms but also to human health. These emerging contaminants have the potential to disrupt endocrine systems, promote the development of antibiotic-resistant bacteria, and bioaccumulate in the food chain, ultimately leading to unacceptable risks to public health. The inability of current conventional treatment methods to effectively remove APIs from water has raised serious concerns about the safety and reliability of water supplies. This issue requires immediate attention and the development of more effective treatment technologies to safeguard the quality of water resources and protect both aquatic ecosystems and human health. Other treatment methods, such as nanotechnology, microalgal treatment, and reverse osmosis, are promising in addressing the issue of API contamination in water resources. These innovative approaches have demonstrated higher removal efficiencies for a wide range of APIs compared to conventional methods, such as activated sludge and chlorination, which have been found to be inadequate in the removal of these emerging contaminants. The potential of these alternative treatment technologies to serve as effective tertiary treatment. To address this critical challenge, governments and policymakers should prioritize investment in research and development to establish effective and scalable solutions for eliminating APIs from various water sources. This should include comprehensive studies to assess the performance, cost-effectiveness, and environmental sustainability of emerging treatment technologies. The emerging contaminants should be included in robust water quality monitoring programs (Aus der Beek et al. in Environ Toxicol Chem 2016;35(4):823-835), with strict regulatory limits enforced to protect public health and the environment. By doing so, the scientific community and regulatory authorities can work together to develop a multi-barrier approach to safeguarding the water resources and ensuring access to safe, clean water for all. This review explores the potential of alternative treatment technologies to serve as viable solutions in the fight against API contamination. Innovative approaches, including nanotechnology, microalgal treatment, and reverse osmosis, have demonstrated remarkable success in addressing this challenge, exhibiting higher removal efficiencies compared to traditional methods.

**Graphical Abstract:**

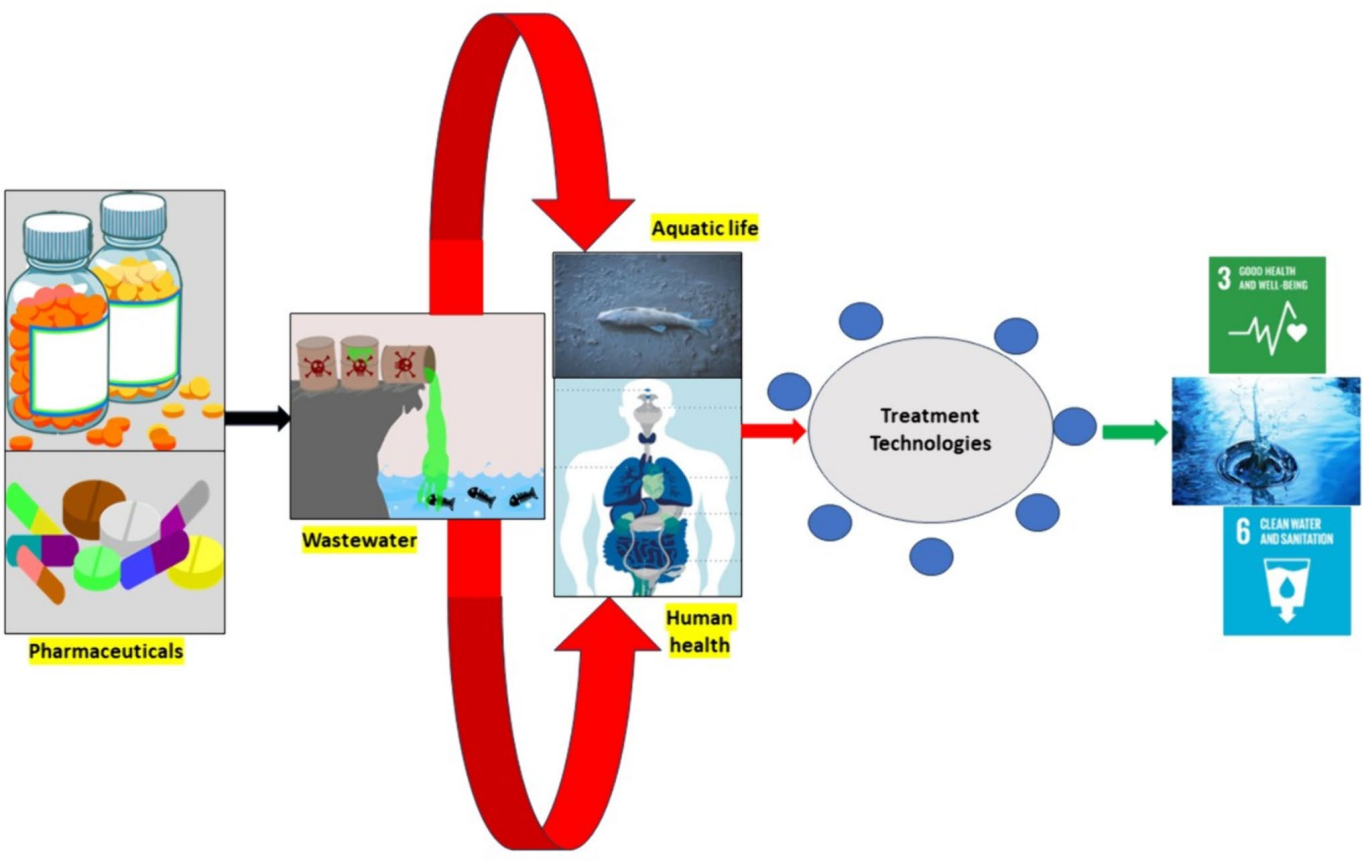

## Introduction

Climate change, urbanisation, water pollution and an increasing population have impacted water availability worldwide. In many parts of the world, the availability of safe water is a significant challenge. Consequently, the importance of preserving or maintaining water bodies clean should be emphasised [1, 2]. South Africa is classified as a semi-arid country, and it is postulated that by 2030, the country will have a water deficit of 17% [3]. Water reclamation and reuse through direct potable reuse have been proposed as measures to combat water scarcity in South Africa [4]. Reclaimed water can be used for irrigating agricultural land, as a source of potable water, for industrial processes, and for groundwater replenishment.

Water resources around the world face a variety of contamination challenges, from industrial effluents and agricultural runoff to microplastics and heavy metals [5]. While these represent significant threats to aquatic ecosystems and human health, one emerging class of contaminants has garnered growing attention in recent years—active pharmaceutical ingredients (APIs). Apart from social acceptability and sewage management problems [6], emerging pollutants of concern, namely, pharmaceutical chemicals and personal care products [7], threaten the possibility of such advancements aimed at addressing water shortages. While the implementation of water recycling and reuse technologies holds promise, the presence of emerging contaminants, such as pharmaceutical chemicals and personal care products, poses a significant threat to the safety and viability of these initiatives [7]. The detection of these active pharmaceutical ingredients (APIs) in water bodies, groundwater, and reclaimed water has raised concerns about their potential long-term impacts on human health and aquatic ecosystems [6].

The use of reclaimed water for irrigation, industrial processes, and groundwater replenishment offers the potential to alleviate pressure on freshwater sources and contribute to a more sustainable and resilient water management approach. However, the successful implementation of these water reuse initiatives is contingent on addressing the challenges posed by emerging contaminants, particularly active pharmaceutical ingredients (APIs) and personal care products [6, 7].The presence of APIs in water bodies, groundwater, and reclaimed water has been widely documented in numerous studies, raising concerns about their potential long-term impacts on human health and aquatic ecosystems [6]. These pharmaceutically active compounds, ranging from antibiotics and anti-inflammatory drugs to hormones and antidepressants, can persist in the environment and potentially disrupt endocrine systems, promote the development of antibiotic-resistant bacteria, and accumulate in the food chain [7].

Addressing the issue of API contamination in water resources is crucial, as it threatens the viability and safety of water reclamation and reuse initiatives, which are essential to mitigating water scarcity. This review aims to provide a comprehensive assessment of the current state of knowledge regarding the presence of APIs in drinking water sources, exploring whether this issue is a myth or a reality. By synthesizing evidence from various studies, the review will examine the prevalence of APIs in water, the effectiveness of conventional treatment methods, and the potential application of innovative green technologies for the sustainable removal of these emerging contaminants.

This review present key research gaps and propose future strategies to address the challenge of API contamination in drinking water, aligning with the scope and objectives of this journal. This includes exploring the environmental fate and impacts of APIs, evaluating the efficacy and scalability of green treatment solutions, and informing policy and regulatory frameworks to ensure the protection of public health and the environment. This review aims to contribute to the development of effective strategies and technologies to safeguard the quality of water resources, ultimately supporting the broader goal of achieving sustainable water management and ensuring access to safe, clean water for all.

## Pharmaceutical contaminants in wastewater: sources, environmental implications, and treatment challenges

Pharmaceutical compounds are designed to improve health by preventing or treating diseases. However, upon their intake, these compounds are excreted in their original form or with slight modifications to their structure [8]. When monitoring process effluent from pharmaceutical companies, it is critical to consider the source of such effluents before mapping out the treatment regime required. Various processes are used in the production of pharmaceutical products. These include fermentation, extraction, chemical synthesis, mixing and formulation. The effluents from pharmaceutical production processes, the wastewater generated by pharmaceutical research and development activities must also be considered as a potential source of API contamination. The diverse range of compounds used and tested in these research settings can be discharged into wastewater streams, further contributing to the presence of APIs in the water bodies. In fermentation, enzymes or microorganisms such as bacteria, fungi, yeast and mould are used in the manufacturing process to initiate reactions [9, 10]. In the extraction pathway, chemical and physical extraction techniques are used to remove the active ingredients from the raw materials, including roots, leaves and animal glands. The methods of extraction include,solvent extraction, distillation, pressing and subliming [11]. In chemical synthesis, pharmaceutical products are produced from a single or a series of chemical reactions. Mixing and formulation entail combining various ingredients to create products such as tablets, ointments, medicinal powders and suspensions. Pharmaceutical wastewater is either discharged directly into water bodies (direct dischargers) such as rivers or to wastewater treatment works (indirect dischargers) [9].

**Fig. 1:**
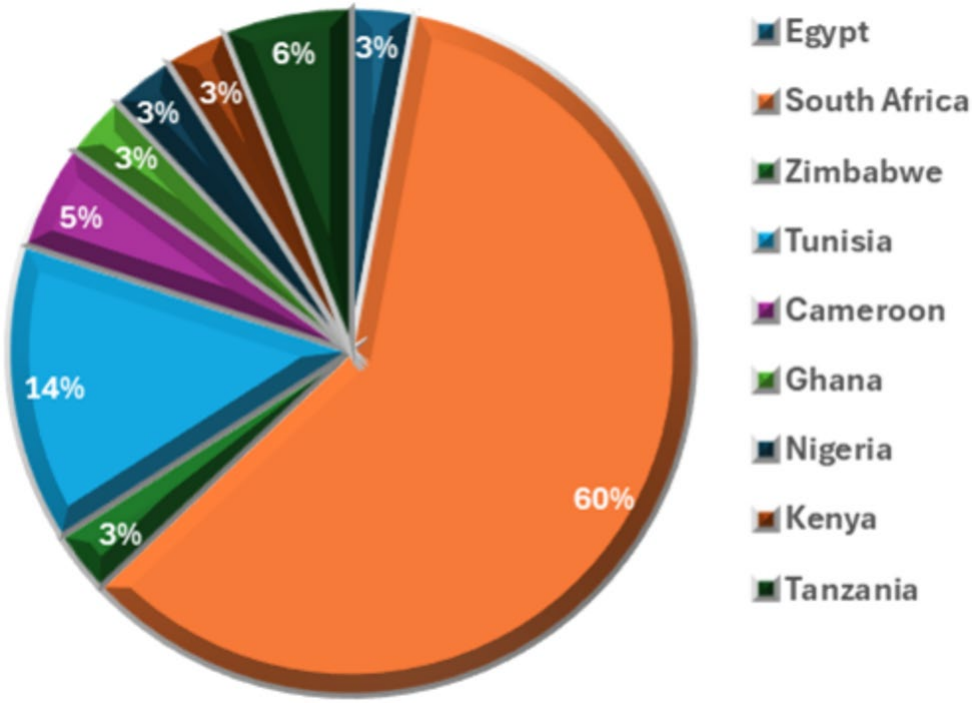
Share of published papers across various African countries on pharmaceuticals in the environment [15]

Generally, wastewater treatment is critical to protect aquatic animals and surface waters typically used as potable water. Conventional wastewater treatment encompasses primary and secondary treatment and mainly aims to remove sediments, their associated pollutants and microorganisms, while chemicals are not targeted [12]. Primary treatment includes the removal of large objects using screens, grit removal and removal of particulate matter in sedimentation tanks. Secondary treatment involves biological processes in a trickling filter or aeration tank, where bacteria remove approximately 85% of the organic matter [13]. This stage is followed by disinfection using chlorine, ozone or ultraviolet light [13]. Pharmaceutical products are increasingly being detected in aquatic systems, soils, groundwater, and biota [14]. They are now listed among contaminants of emerging concern (CEC) which are being widely researched in developed countries [14]. Across Africa, South Africa has the most articles published on this topic [15], as shown in Fig. 1.

CECs are not routinely monitored, although future regulations may include them depending on their environmental impact and public opinions [7].

**Fig. 2:**
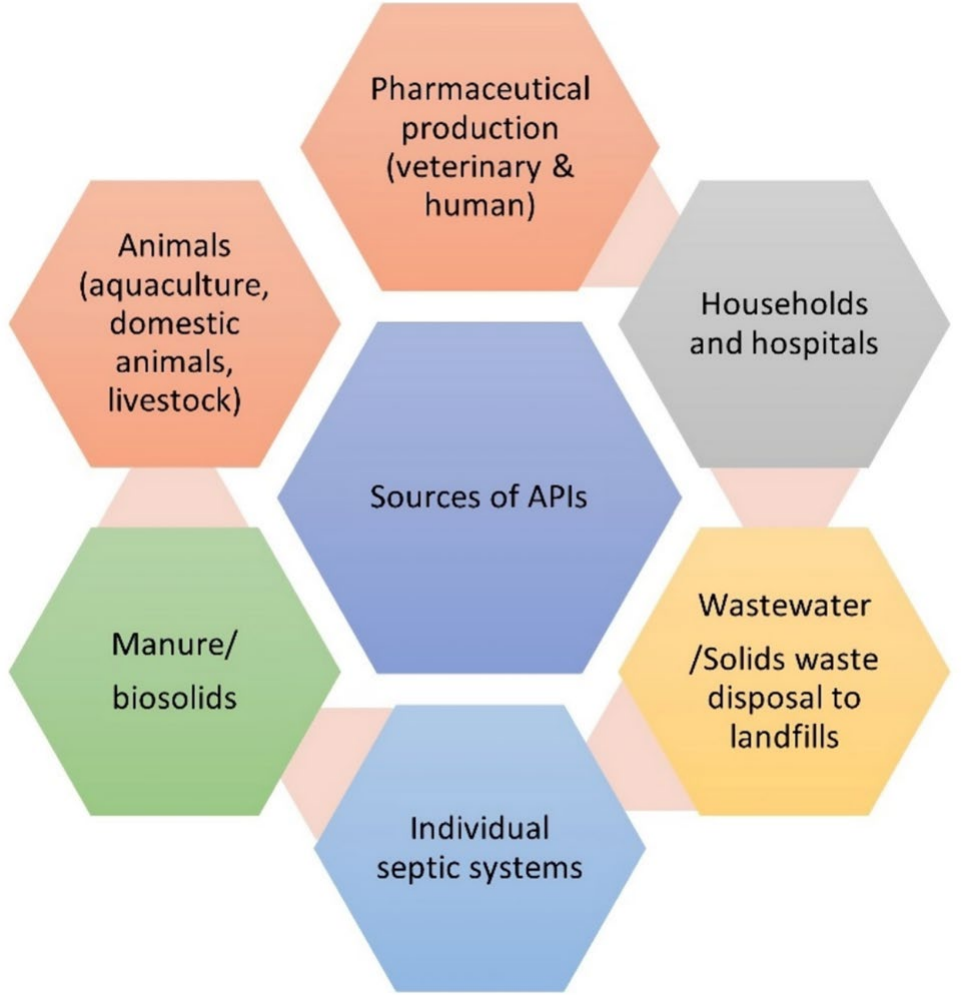
Sources of Pharmaceutical contaminants. Adapted from (OECD [14])

The management of pharmaceutical compounds provides a conundrum due to the many potential sources of such compounds, as illustrated in Fig. 2. Therefore, targeting pharmaceutical industries or wastewater treatment plants alone is inadequate in controlling the ingress of these compounds into the environment. These compounds have also been detected in the pristine Antarctica and Arctic regions [16]. It is reported that around 3 000 pharmaceutical substances enter wastewater treatment plants yearly, with evidence of their prevalence reported in several countries globally, including Brazil, Canada, France, China, Sweden and South Africa, as cited by [12].

In areas with poor sanitation, human excreta are disposed of on the ground or surface waters. When there is rain, pharmaceutical compounds are washed off into the water. Other potential sources include leaking septic tanks, flushing unused medicines, landfill leachates, excreta from animals and wastewater treatment plant sludge used for agricultural purposes [8, 17]. Pharmaceutical compounds are given to animals orally, topically or in the feed. These include antibiotics, deworming medicines, insecticides, and growth hormones [16].

According to Patel et al. [16], the following is the order of concentration of pharmaceutical compounds: Industrial effluents > hospital effluents > wastewater treatment plant effluents > surface water > groundwater > drinking water. Similar to the perceived harmful effects of plastic waste on humans and marine life, pharmaceuticals can be resistant to degradation, persistent pollutants in water bodies, lipophilic or water-soluble, and can be taken up by biota and bioaccumulate. Pharmaceutical contaminants pose a threat not only to plants and aquatic animals but also humans upon consumption or on drinking water. Pharmaceuticals cannot be classified under a single category like other known pollutants, such as chlorofluorocarbons (CFCs), since they vary in physicochemical properties, chemical structure, and biological properties. Pharmaceutical compounds are designed to target specific organisms; numerous compounds are polar. Non-steroidal anti-inflammatory drugs (NSAIDs) such as naproxen and antibiotics, sulfamethoxazole, and erythromycin can remain in the environment for about one year; other pharmaceuticals can persist for many years, while halogenated drugs are more stable in the environment [16]. Another problem with pharmaceutical contaminants is their ability to adsorb and subsequent distribution in living organisms. These compounds can be transformed metabolically, resulting in structure modifications [16].

The concentration of pharmaceutical contaminants is also dependent on the region and season. For example, the risk of finding these emerging pollutants in regions where drug usage is limited is reduced. In winter, antibiotic consumption increases due to the prevalence of upper respiratory infections leading to a higher concentration of these compounds in wastewater influents. Consumption patterns of drugs used as cough medicines, such as ephedrine, pseudoephedrine, and pholcodine, also increase during winter. At the same time, in other instances, pandemics are responsible for increased active pharmaceutical ingredients in wastewater. For example, the HIV/AIDS pandemic has led to the production of significant amounts of antiretrovirals, while the advent of Covid-19 resulted in the formulation of various vaccines. Therefore, it is necessary to monitor the presence of these drugs in aquatic systems and drinking water and study their effects on humans and biota.

Over-the-counter medicines such as NSAIDs will likely be found in higher environmental concentrations, unlike prescribed medication [8]. In the environment, pharmaceuticals can be transformed into either more or less toxic compounds. For example, drugs, such as aspirin, dissociate to carbon dioxide and water. Some compounds are adsorbed by the sludge in wastewater treatment plants, while some are bonded in matrices within the environment. Other compounds become hydrophilic and eventually enter receiving waters as they escape from treatment plants. Transformation of pharmaceuticals is initiated by partitioning, volatilisation, hydrolysis, microbial activity and photodegradation. Some removal efficiencies have been tabulated for conventional and advanced wastewater treatment processes, as shown in Tables 1 and 2 adopted from Osunmakinde et al. [12]. These efficiencies depend on the nature of the substance, season and the wastewater treatment plant.

**Table 1:** Conventional wastewater treatment [12]

Type	Removal range (%)	Source	Country	Pros	Cons
Activated sludge	11–99	Raw sewage	Australia	Relatively effective for a wide range of APIs [18]	Can be variable and incomplete removal [19], requires significant energy and infrastructure [20]
	7–100	Primary settled sewage	EU, Japan		
	< 20–80	Primary settled sewage	France		
	8–98	-	Brazil, EU, Japan		
Biological filtration	6–71	Primary settled sewage	Europe	Can be effective for some APIs [21], relatively simple [22]	Less effective for certain polar and persistent APIs [21]
Primary settling	3–45	Not indicated	Brazil, EU, Japan	Simple, low-cost [23]	Very limited removal of APIs [21]
Coagulation filtration and settling	5–36	Not indicated		Removes some APIs [24], can be combined with other treatments	Moderately effective [24], additional energy and chemical inputs required [20]
Sand filtration	0–99	Activated sludge effluent		Can achieve high removal for some APIs [22]	Highly variable and dependent on filter design and operation [24]

**Table 2:** Advanced wastewater treatment [12]

Type	Removal range (%)	Source	Country	Pros	Cons	Effective Mechanism
Ozonation	1–99% 86–100	Activated sludge effluent Secondary effluent	Brazil, EU, Japan	Highly effective for a wide range of APIs [25, 26]	Can generate potentially toxic transformation products [27]	Oxidation
Ozonation/ultrasound and sonocatalysis	23–45	Not indicated	EU, India, Japan, Turkey, USA	Can enhance API removal compared to ozonation alone [28, 29]	Relatively moderate removal efficiencies [28, 29]	Oxidation, Degradation
Ozonation and catalytic ozonation	> 9–100	-		Improved removal compared to ozonation alone [30]	Can be energy-intensive [30]	Oxidation
UV irradiation	29	Not indicated	Brazil, EU, Japan	Can degrade some APIs [31]	Limited effectiveness for many APIs [31]	Photolysis, Degradation
Photolysis hydrogen peroxide	52–100	Not indicated	EU, India, Japan, Turkey, USA	Effective for degrading a range of APIs [32, 33]	Additional chemical and energy inputs required [32, 33]	Oxidation, Free Radicals
Dark and light Fenton	80–100			High removal of many APIs [30]	Requires careful control of pH and other parameters [30]	Oxidation, Free Radicals
UV/TiO_2_	> 95			Excellent removal of various APIs [31]	Complex and energy-intensive process [31]	Photocatalysis, Oxidation
Biomembrane	23–99	Treated effluent	Brazil, EU, Japan	Can effectively remove some APIs [34]	Variable and sometimes limited removal [34]	Adsorption, Degradation
Reverse osmosis	62–100	Secondary treated effluent	Not indicated	Highly effective for a wide range of APIs [24]	Concentrated waste stream requires further treatment [24]	Separation, Adsorption
Ultrasound	24–100	Not indicated	EU, India, Japan, Turkey	Can enhance API removal when combined with other treatments [28, 29]	Moderate removal when used alone [28, 29]	Cavitation, Degradation
Grit-biological reactor-clarifier-sand filtration-ozonation-biological activation-carbon microfiltration-UV chlorination	> 90	Wastewater	Australia	Comprehensive multi-barrier approach for high API removal [35]	Complex and energy/chemical-intensive treatment train [35]	Multiple Barriers (Oxidation, Adsorption, Degradation)
Grit-activated sludge/UV-microfiltration-reverse osmosis	> 90	Wastewater	Australia	Highly effective API removal [35]	Concentrated waste stream and high energy demands [35]	Multiple Barriers (Oxidation, Adsorption, Separation)
Grit-activated sludge-UV-microfiltration-chlorination-reverse osmosis	> 90	Wastewater	Australia	Comprehensive treatment for effective API removal [35]	Significant infrastructure and operational costs [35]	Multiple Barriers (Oxidation, Adsorption, Separation)

## Potential effects of pharmaceutical compounds in aquatic animals and humans

Overall, even at low exposure levels [7], pharmaceuticals appear to affect the population of aquatic animals by causing disruptions in the reproductive system, affecting the genetic system, which results in poor fitness of the population and decreased survival chances, or in the case of psychiatric drugs such as oxazepam, some organisms become docile and less fearful of predators while others end up as aggressive feeders [36]. This results in ecological disturbances and the excessive growth of algae when the population of zooplankton that feeds on algae is significantly reduced. An overgrowth of algae creates dead zones, which are areas of low oxygen, and fish cannot survive in these regions. Residual antibiotics in aquatic systems can develop pathogens resistant to the current classes of antibiotics [37]. Simultaneously humans are also at risk of developing antibiotic resistance through drinking water from contaminated sources [8]. However, Kostich et al. argue that healthy individuals are less likely to be at risk from active pharmaceutical compounds [37]. Antibiotics present in sludges or farm manure used as fertiliser can also affect root development in plants and hinder plant growth [38, 39]. There is also a possibility of people living with HIV who are not on antiretroviral therapy (ART) developing viral resistance over time. In healthy people, exposure to ARVs could cause nausea and liver and kidney damage [40]. Antiepileptic medicines may lead to developmental problems in humans and aquatic animals and potentially cause disruptions in the endocrine system by reducing thyroid hormones, oestrogen and testosterone and, in turn, decreasing fertility. Adverse effects on the nervous system, which include drowsiness, and problems with vision, have also been reported [41]. It is, therefore key to evaluate the concentrations of these substances in freshwater and marine ecosystems and determine the safe exposure limits.

## Potential treatment processes investigated

Pharmaceutical compounds can be organic and inorganic; therefore, different extraction techniques may need to be investigated. Metallodrugs (inorganic compounds) have been used in treating diseases such as diabetes, wounds, cancer, bacterial and parasitic infections, inflammatory diseases such as arthritis, cardiovascular problems and neurological conditions. These metalloids include platinum, antimony, silver, and vanadium [42, 43]. Under organic pharmaceuticals, it is also crucial to evaluate illicit drugs. Wastewater-based epidemiology (WBE) has been used in various populations to determine exposure levels or consumption patterns of certain chemicals or drugs in an area by analysing wastewater [44]. For example, in China, WBE is used to find companies producing illicit drugs, initiate arrests of the people involved and the subsequent closures of these companies [45]. The first detection of illicit drugs in water bodies was reported by Zucatto et al. [46], the authors detected cocaine and its metabolite from urine, benzoylecgonine in wastewater samples from several plants and the Po River from Italy. Deng et al. [45] collected wastewater samples from several treatment plants in China and analysed 12 illicit drugs. The drugs of interest were methamphetamine (crystal meth. or tik as it is called in South Africa), cocaine, 3,4-methylenedioxymethamphetamine, benzoylecgonine, amphetamine, methadone, norketamine, codeine, ketamine, 3,4-methylenedioxyamphetamine, methcathinone, heroin, and methamphetamine-d8. The most common drug detected was crystal meth. The authors also found various removal rates from wastewater, with the sequential batch reactor, an activated sludge-based technology removing the highest amount of drugs [45].

**Fig. 3:**
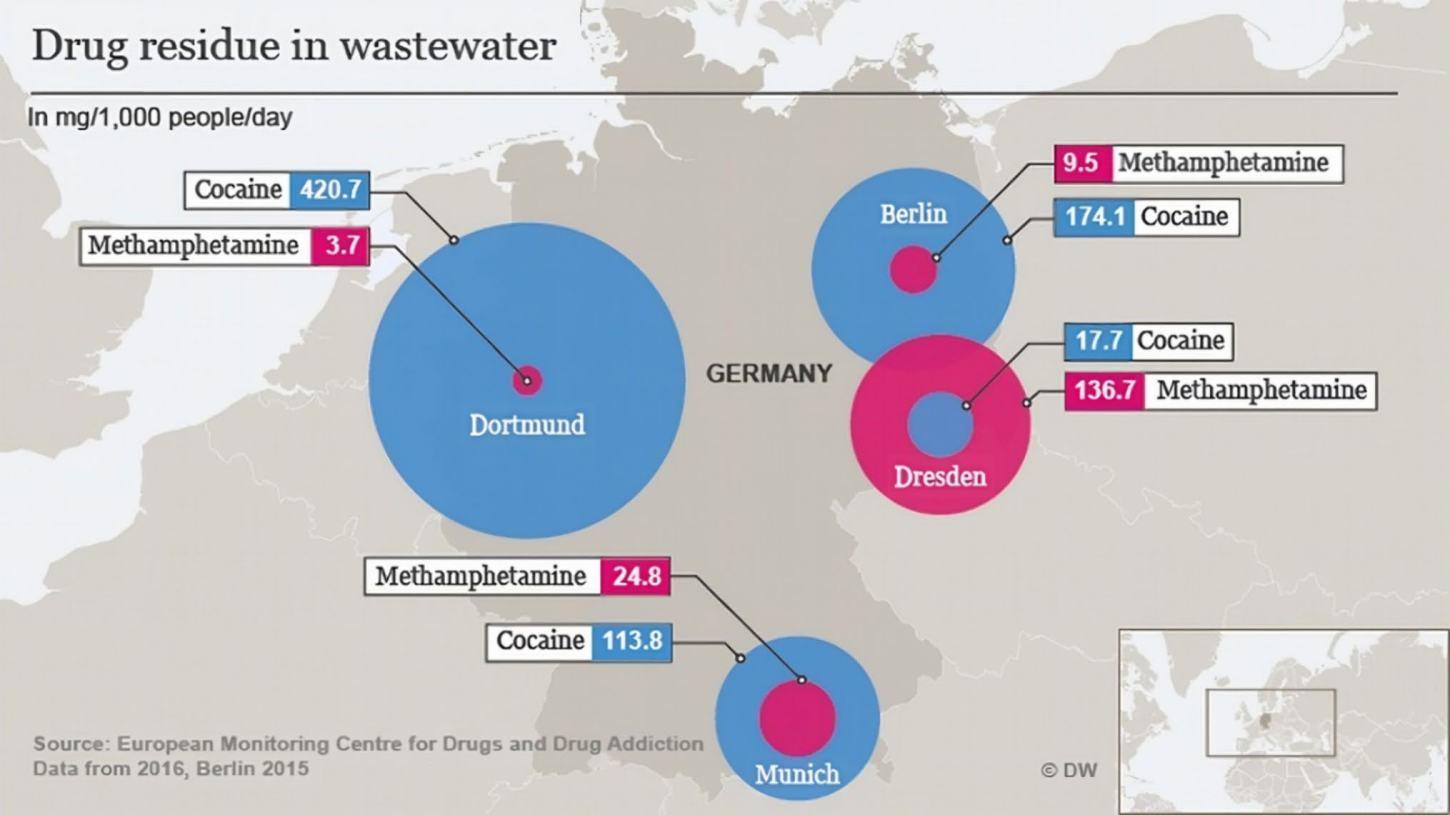
Concentrations of illicit drugs, cocaine, and crystal meth. detected in German wastewater. Adopted from (DW [49])

González-Mariño et al. [47] also conducted research in several regions, including the United States, Australia and South America, between 2011 and 2017. Wastewater from 120 cities in 37 countries was analysed. The authors confirmed benzoylecgonine, amphetamine, ecstasy and crystal meth. (methamphetamine) in various concentrations. In South Africa, Archer et al. [48] conducted WBE studies at two wastewater treatment plants in Gauteng and Western Cape Provinces. The authors found that crystal meth. (tik) was more prevalent in the influent, with concentrations ranging between 181.9 to 1185 mg/day per 1000 people, followed by cocaine and ecstasy, respectively [48]. In Germany, cocaine and crystal meth. were also found in wastewater sampled in Dortmund, Berlin, Munich and Dresden in the concentrations shown in Fig. 3 [49].

Another class of drugs of interest are contrast media. The continued use of medical imaging in diagnosing diseases has also resulted in the increased use of these drugs. Dekker et al. [50] cited numerous authors who have detected iodinated contrast media, commonly used during CT scans, in surface and drinking water [50]. The authors also highlighted that upon disintegration, such media produced toxic by-products. Other contrast media of concern were gadolinium for magnetic resonance imaging and barium for various x-rays.

Brünjes and Hofmann [51] discovered that the increasing use of gadolinium-based contrast agents (GBCAs) for medical imaging is leading to widespread contamination of freshwater and drinking water supplies, with concerns about their degradation and the resulting health impacts, especially where water treatment processes and water sources are involved. Several authors, including Hatje et al. [52], Ebrahimi and Barbieri [53], Tepe et al. [54], Kulaksiz and Bau [55] and Schmidt et al. [56] also detected gadolinium in river water, groundwater, surface water and drinking water. However, no data was found for Africa. In 2016, as much as 19 tonnes, 21 tonnes and 0.3 tonnes of gadolinium were emitted in the European Union, the United States and Switzerland, respectively [51]. There are also reported interactions between organic drugs and contrast media [57–59], which can complicate the treatment regime employed to remove pharmaceuticals from wastewater.

The US.EPA [60] generated a database of treatment technologies used in numerous industries, including the pharmaceutical sector, at pilot and full scale by reviewing peer-reviewed studies, grey literature and conferences released since 2000. Table 3 illustrates the findings of their review study. However, the study did not specify the removal efficiencies of APIs such as anti-inflammatories, anti-diabetes, antihistamines, and anticonvulsants. Other technologies that have been highlighted include phytoremediation, electro-coagulation combined with electroflotation, use of nanocomposites as adsorbents or molecularly imprinted polymers [8].

**Table 3:** Treatment Technologies for the removal of active pharmaceutical ingredients (APIs) from pharmaceutical industry wastewater [60]

Main treatment technology	Technology combinations	Pilot or Full scale	Parameter	Efficiency (%)	Cited reference
Flow Equalisation	EQ ➝ ANSG ➝ BASR	Pilot	Ampicillin	50	ASCE, 2006 [61]
	EQ ➝ ANSG ➝ BASR	Pilot	Chemical oxygen demand	98.18	
	EQ ➝ ANSG ➝ BASR	Pilot	Chlortetracycline (Aureomycin)	36.67	
	MPT ➝ EQ ➝ BIO ➝ CLAR ➝ MBBR ➝ ASG ➝ CLAR	Full	Chemical oxygen demand	> 80	State Key Laboratory of Pollution Control and Resource Reuse, School of the Environment, Nanjing University, China, 2010
	MPT ➝ EQ ➝ BIO ➝ CLAR ➝ MBBR ➝ ASG ➝ CLAR	Full	Ammonium-nitrogen (NH_4_-N)	76.85	
	EQ ➝ MBR	Full	Ammonia (as N)	95.71	Hatch Mott MacDonald, Iselin, New Jersey, 2013
	EQ ➝ MBR	Full	Chemical oxygen demand	91.56	
	EQ ➝ MBR	Full	Nitrite Plus Nitrate Total	-	
	EQ ➝ MBR	Full	Nitrogen, Kjeldahl total (TKN)	84.18	
	EQ ➝ MBR	Full	Total Dissolved Solids (TDS)	-	
	EQ ➝ AD ➝ MBR ➝ OZ ➝ DGS	Full	Active Pharmaceutical Ingredients (APIs)	100	Xylem Water Solutions, Germany, 2014
	EQ ➝ AD ➝ MBR ➝ OZ ➝ DGS	Full	Biological Oxygen Demand	98.91	
	EQ ➝ AD ➝ MBR ➝ OZ ➝ DGS	Full	Chemical oxygen demand	98.03	
	EQ ➝ AD ➝ MBR ➝ OZ ➝ DGS	Full	Estrogenicity, 17-beta estradiol equivalent Estrogenicity, 17-beta estradiol equivalent Nitrogen, organic	99.92	
Membrane Bioreactor	MBR	Pilot	-	-	Dewberry, 2012
	MBR	Pilot	-	-	
	EQ ➝ MBR	Full	Ammonia (as N)	95.71	Hatch Mott MacDonald, Iselin, New Jersey, 2013
	EQ ➝ MBR	Full	Chemical oxygen demand	91.56	
	EQ ➝ MBR	Full	Nitrite Plus Nitrate Total	-	
	EQ ➝ MBR	Full	Nitrogen, Kjeldahl total (TKN)	84.18	
	EQ ➝ MBR	Full	Total Dissolved Solids (TDS)	-	
	EQ ➝ AD ➝ MBR ➝ OZ ➝ DGS		Active Pharmaceutical Ingredients (APIs)	100	Xylem Water Solutions, Germany, 2014
	EQ ➝ AD ➝ MBR ➝ OZ ➝ DGS		Biological Oxygen Demand	98.91	
	EQ ➝ AD ➝ MBR ➝ OZ ➝ DGS		Chemical oxygen demand	98.03	
	EQ ➝ AD ➝ MBR ➝ OZ ➝ DGS		Estrogenicity, 17-beta estradiol equivalent Estrogenicity, 17-beta estradiol equivalent Nitrogen, organic	99.92	
Aerobic Biological Treatment	**AD ➝ MF**	Full	-	-	Praxair, Inc. 7000 High Grove Boulevard, Burr Ridge, IL, USA, 2013
	EQ ➝ AD ➝ MBR ➝ OZ ➝ DGS	Full	Active Pharmaceutical Ingredients (APIs)	100	Xylem Water Solutions, Germany, 2014
	EQ ➝ AD ➝ MBR ➝ OZ ➝ DGS	Full	Biological Oxygen Demand	98.91	
	EQ ➝ AD ➝ MBR ➝ OZ ➝ DGS	Full	Chemical oxygen demand	98.03	
	EQ ➝ AD ➝ MBR ➝ OZ ➝ DGS	Full	Estrogenicity, 17-beta estradiol equivalent Estrogenicity, 17-beta estradiol equivalent Nitrogen, organic	99.92	
Moving Bed Bioreactor	MPT ➝ EQ ➝ BIO ➝ CLAR ➝ MBBR ➝ ASG ➝ CLAR	Full	Chemical oxygen demand	> 80	State Key Laboratory of Pollution Control and Resource Reuse, School of the Environment, Nanjing University, China, 2010
	MPT ➝ EQ ➝ BIO ➝ CLAR ➝ MBBR ➝ ASG ➝ CLAR	Full	Ammonium-nitrogen (NH_4_-N)	76.85	
	MBBR ➝ MBBR ➝ DAF	Full	Chemical oxygen demand	57.24	Aqwise—Wise Water Technologies Ltd, 2018
Anaerobic Suspended Growth	EQ ➝ ANSG ➝ BASR	Pilot	Ampicillin	50	ASCE, 2006 [61]
	EQ ➝ ANSG ➝ BASR	Pilot	Chemical oxygen demand	98.18	
	EQ ➝ ANSG ➝ BASR	Pilot	Chlortetracycline (Aureomycin)	36.67	
Aerobic Suspended Growth	MPT ➝ EQ ➝ BIO ➝ CLAR ➝ MBBR ➝ ASG ➝ CLAR	Full	Chemical oxygen demand	> 80	State Key Laboratory of Pollution Control and Resource Reuse, School of the Environment, Nanjing University, China, 2010
	MPT ➝ EQ ➝ BIO ➝ CLAR ➝ MBBR ➝ ASG ➝ CLAR	Full	Ammonium-nitrogen (NH_4_-N)	76.85	
Biofilm Airlift Suspension Reactor	EQ ➝ ANSG ➝ BASR	Pilot	Ampicillin	50	ASCE, [61]
	EQ ➝ ANSG ➝ BASR	Pilot	Chemical oxygen demand	98.18	
	EQ ➝ ANSG ➝ BASR	Pilot	Chlortetracycline (Aureomycin)	36.67	
Clarification	MPT ➝ EQ ➝ BIO ➝ CLAR ➝ MBBR ➝ ASG ➝ CLAR	Full	Chemical oxygen demand	> 80	State Key Laboratory of Pollution Control and Resource Reuse, School of the Environment, Nanjing University, China, 2010
	MPT ➝ EQ ➝ BIO ➝ CLAR ➝ MBBR ➝ ASG ➝ CLAR	Full	Ammonium-nitrogen (NH_4_-N)	76.85	
Dissolved Air Flotation	MBBR ➝ MBBR ➝ DAF	Full	Chemical oxygen demand	57.24	Aqwise—Wise Water Technologies Ltd, 2018
Degasification	EQ ➝ AD ➝ MBR ➝ OZ ➝ DGS	Full	Active Pharmaceutical Ingredients (APIs)	100	Xylem Water Solutions, Germany, 2014
	EQ ➝ AD ➝ MBR ➝ OZ ➝ DGS	Full	Biological Oxygen Demand	98.91	
	EQ ➝ AD ➝ MBR ➝ OZ ➝ DGS	Full	Chemical oxygen demand	98.03	
	EQ ➝ AD ➝ MBR ➝ OZ ➝ DGS	Full	Estrogenicity, 17-beta estradiol equivalent Estrogenicity, 17-beta estradiol equivalent Nitrogen, organic	99.92	
Micro- and Ultra-Membrane Filtration	**AD ➝ MF**	Full	-	-	Praxair, Inc. 7000 High Grove Boulevard, Burr Ridge, IL, USA, 2013
Mechanical Pre-Treatment	MPT ➝ EQ ➝ BIO ➝ CLAR ➝ MBBR ➝ ASG ➝ CLAR	Full	Chemical oxygen demand	> 80	State Key Laboratory of Pollution Control and Resource Reuse, School of the Environment, Nanjing University, China, 2010
	MPT ➝ EQ ➝ BIO ➝ CLAR ➝ MBBR ➝ ASG ➝ CLAR	Full	Ammonium-nitrogen (NH_4_-N)	76.85	
Ozonation	EQ ➝ AD ➝ MBR ➝ OZ ➝ DGS	Full	Active Pharmaceutical Ingredients (APIs)	100	Xylem Water Solutions, Germany, 2014
	EQ ➝ AD ➝ MBR ➝ OZ ➝ DGS	Full	Biological Oxygen Demand	98.91	
	EQ ➝ AD ➝ MBR ➝ OZ ➝ DGS	Full	Chemical oxygen demand	98.03	
	EQ ➝ AD ➝ MBR ➝ OZ ➝ DGS	Full	Estrogenicity, 17-beta estradiol equivalent Estrogenicity, 17-beta estradiol equivalent Nitrogen, organic	99.92	

*EQ*; Equalization, *ANSG*; Anaerobic sludge digestion, *BASR*; Batch activated sludge reactor, *MPT*; Multi-phase treatment, *BIO*; Biological treatment, *CLAR*; Clarification, *MBBR*; Moving bed biofilm reactor, *ASG*; Activated sludge, *MBR*; Membrane bioreactor, *AD*; Adsorption, *OZ*; Ozonation, *DGS*; Disinfection/gas stripping, *MF*; Microfiltration, *DAF*; Dissolved air flotation

Other typical removal efficiencies have been reported by Osunmakinde et al. [12], as shown in Table 4 with the majority of studies reviewed focussing on advanced oxidation processes including ozonation, ultraviolet (UV), photolysis. Fenton processes. de Salles Pupo et al. [62] reviewed various green treatment methods, including advanced oxidation processes (AOPs), adsorption using biosorbents, membrane filtration, and biological treatment approaches. These technologies leverage eco-friendly materials and renewable energy sources to degrade or remove pharmaceutical compounds. The paper highlighted the advantages of green technologies, such as their high efficiency, low energy consumption, and reduced environmental impact compared to conventional wastewater treatment methods. It also discusses the challenges and limitations associated with implementing these emerging technologies at scale. Lin et al. [63] reported the removal efficiencies for caffeine and sulfamethoxazole with advanced oxidation processes as 89.5% and 92.2% respectively. Paredes et al. [64] observed high rates of photodegradation for diclofenac and diazepam using UV treatment. However, Adams et al. [65] observed poor removal efficiency of antibiotics using UV treatment. Overall, assessing treatment efficiencies presents a challenge as these are also dependent on operating parameters such as pH and temperature and other variables including total suspended solids and total dissolved solids [62]. UV treatments have also been questioned as they can potentially break down contrast media such as gadolinium into toxic compounds [51].

**Table 4:** Removal efficiencies in advanced treatment processes [12]

Type	Removal range (%)	Source	Country	Effective Mechanism
Ozonation	1–99%	Activated sludge effluent	Brazil, EU, Japan	Oxidation
	86–100	Secondary effluent		Oxidation, Degradation
Ozonation/ultrasound and sonocatalysis	23–45	Not indicated	EU, India, Japan, Turkey, USA	Oxidation
Ozonation and catalytic ozonation	> 9–100	-		Oxidation
UV irradiation	29	Not indicated	Brazil, EU, Japan	Photolysis, Degradation
Photolysis hydrogen peroxide	52–100	Not indicated	EU, India, Japan, Turkey, USA	Oxidation, Free Radicals
Dark and light Fenton	80–100			Oxidation, Free Radicals
UV/TiO_2_	> 95			Photocatalysis, Oxidation
Biomembrane	23–99	Treated effluent	Brazil, EU, Japan	Adsorption, Degradation
Reverse osmosis	62–100	Secondary treated effluent	Not indicated	Separation, Adsorption
Ultrasound	24–100	Not indicated	EU, India, Japan, Turkey	Cavitation, Degradation
Grit-biological reactor-clarifier-sand filtration-ozonation-biological activation-carbon microfiltration-UV chlorination	> 90	Wastewater	Australia	Multiple Barriers (Oxidation, Adsorption, Degradation)
Grit-activated sludge/UV-microfiltration-reverse osmosis	> 90	Wastewater	Australia	Multiple Barriers (Oxidation, Adsorption, Separation)
Grit-activated sludge-UV-microfiltration-chlorination-reverse osmosis	> 90	Wastewater	Australia	Multiple Barriers (Oxidation, Adsorption, Separation)

The removal efficiency of treatment technologies for removing APIs from wastewater is typically defined as the percentage of the initial API concentration that is removed or reduced after the treatment process. This is calculated by measuring the influent (before treatment) and effluent (after treatment) concentrations of the APIs using analytical techniques such as high-performance liquid chromatography (HPLC), liquid chromatography-mass spectrometry (LC–MS), or gas chromatography-mass spectrometry (GC–MS), and then applying equation

1 $$Removal\;efficiency\;\left(\%\right)=\left[\frac{C_{in-C_{out}}}{C_{in}}\right]\times100$$

where C_in_ is the influent concentration and C_out_ is the effluent concentration. The removal efficiency can be influenced by various factors, including the characteristics of the APIs, the design and operational parameters of the treatment technologies, the composition and characteristics of the pharmaceutical industry wastewater, and the presence of other contaminants or matrix effects. By calculating and reporting the removal efficiency, researchers and engineers can assess the performance of different treatment technologies or combinations of technologies in removing APIs from pharmaceutical industry wastewater and make informed decisions about the most effective treatment strategies.

## Pollutants in Africa’s wastewater

The most prescribed medicines in South Africa are illustrated in Table 5 [12]**.** Due to their prevalence in usage, these drugs are highly likely to be detected in South Africa’s wastewater, drinking water, or groundwater.

**Table 5:** Most prescribed medicines in South Africa [12]

Drug	Drug Type
Paracetamol	Analgesic
Albendazole	Antihelmintic
Chlorphenoxamine hydrochloride	Anti-Allergic
Chloramphenicol;Amoxycillin; Ampicillin; Ceftriaxone	Antibiotics
Hydrocortisone acetate	Corticosteroid
Lamivudine; Efavirenz; Stavudine; Tenofovir	ARV
Salbutamol Sulphate	Asthma
Simvastatin	Cholesterol
Levonorgestrel and Ethinyloestradiol; Norgestrel; Norethisterone	Contraceptives
Cocillana	Cough syrup
Metformin Hydrochloride; Gliclazide; Insulin	Diabetes
Hydrochlorothiazide; Enalapril maleate, Amlodipine, Nifedipine; Perindopril	Hypertension
Methyl salicylate	NSAID
Atenolol	β-blocker

In the review study conducted by Madikizela et al. [8] to determine the milestones made in pharmaceutical pollution of water bodies in Africa, the authors found that the NSAIDs,naproxen, ibuprofen, diclofenac and ketoprofen were prevalent in wastewater and surface water, with concentrations higher than those reported for high-income countries in Europe. The researchers concluded that this could be attributed to inadequate sanitation and poor removal efficiencies of wastewater treatment plants in Africa [8]. In South Africa’s rivers, such as Umgeni and Mbokodweni, the NSAIDs mentioned above and paracetamol were detected. Antibiotics were also detected in surface waters and wastewater. However, their concentrations in surface waters were reportedly lower compared to NSAIDs. In a 2011 study by [66], the authors collected samples downstream of 10 sewage treatment plants and analysed the presence of cardiovascular, analgesic, anti-inflammatory and antipyretic pharmaceuticals. Of the 24 compounds assessed, 21 had concentrations between 2 ng L^−1^ to 18 μg L^−1^ and this included ibuprofen (NSAID), diclofenac (NSAID), naproxen, atenolol, frusemide, gemfibrozil, and hydrochlorothiazide. However, no pharmaceuticals from the assessed groups were found in the drinking water samples collected.

Another group of pharmaceuticals detected were antiretrovirals (ARVs) Madikizela et al. [8]. South Africa boasts the most extensive HIV treatment programme in the world, with approximately 5.4 million people (9% of the population) accessing ARV treatment. Therefore, levels of ARVs in wastewater, surface water and drinking water are critical to monitor. Antiepileptics such as carbamazepine were also detected in aquatic systems and drinking water while steroidal hormones detected in South Africa’s wastewaters and surface waters included estrone, 17-β-estradiol, estriol, 17-α–ethinylestradiol, progesterone and testosterone. Waleng and Nomngongo [15] reviewed similar studies and found that antibiotics and NSAIDs were prevalent in Africa’s wastewater. Mhuka et al. [67] conducted experimental work to evaluate various pharmaceutical compounds in influent and effluent from a Wastewater Treatment Facility in Pretoria, South Africa. Antibiotics were the primary compounds in influent, constituting about 28%. Other compounds detected were non-steroidal anti-inflammatory drugs (NSAIDs), steroid hormones, antifungal and antimicrobial compounds. Mhuka et al.’s findings generally agree with results from other authors including Papageorgiou et al., [68], who observed that NSAIDs, antibiotics, anti-hypertensives and psychiatric drugs were common in wastewater.

Mhuka et al. [67] categorized the removal efficiencies of emerging contaminants into three groups, as illustrated in Table 6. These categories are: negative removal, 0–70% removal, and above 70% removal. Negative removal indicates that the contaminant concentration increased in the effluent, rather than being reduced.. According to Ternes et al. [22], when the solid–liquid partition coefficient, K_d_ is less than 500 l/kg, a compound’s sorption efficiency onto sludge during the wastewater treatment process is lowered, hence the varying degrees of removal efficiency. Removal rates were also not dependent on the compound’s classification. The authors concluded that wastewater treatment plants are currently not suited for the complete removal of contaminants associated with pharmaceutical and personal care products wastewater. Concentration increase has been attributed to possible transformations or accumulation in the process [67].

**Table 6:** Pharmaceutical group, drug type and removal efficiency from wastewater (adapted from (Mhuka et al. [67]))

Group	Negative removal	0–70% removal	Over 70% removal
Antidepressants	Amitriptyline, venlafaxine	-	-
Antiretrovirals (ARVs)	Atazanavir, nevirapine, ritonavir	Efavirenz	Lamivudine
Anthelmintics	-	Mebendazole	Albendazole
Anti-bacterial and anti-fungal agents	Triclosan	Fluconazole	Sulfapyridine, triclocarban
Anticonvulsants (anti-epileptic)	Carbamazepine, gabapentin	-	-
Antibiotics	Clarithromycin, isoniazid (TB), lincomycin, ofloxacin, oxolinic acid, sulfadimethoxine, sulfamethazine, sulfanilamide	Sulfamethoxazole, trimethoprim	Ciprofloxacin, enrofloxacin, flumequine, erythromycin, norfloxacin, oxytetracycline, sarafloxacin, sulfadiazine, sulfaguanadin
Analgesics	Tramadol	-	Paracetamol, salicylamide
*Hormones	Diethylstilbestrol (synthetic estrogen), estradiol and estriol (sex hormones)	Medroxyprogesterone, mestranol, progesterone	Testosterone
Angiotensin-converting enzyme inhibitors (blood pressure), Calcium channel blockers	Enalapril, verapamil	Valsartan	-
Antivirals	Famciclovir	Penciclovir	-
Antifungal	Thiabendazole	-	-
Chemotherapy	Ifosfamide	-	
Non-steroidal anti-inflammatory drugs (NSAIDs)	Ketoprofen, naproxen, phenacetin, Bufexamac, mefenamic acid	Diclofenac, indomethacin	Ibuprofen
Anaesthetic	Lidocaine	-	-
Beta-blockers	metoprolol	-	Pindolol
Corticosteroids	Predinisolone	-	-
Adrenergic bronchodilators	Salbutamol	Terbutaline	-
Fibrates	Gemfibrozil	-	-
Adrenergic agonists	-	Ractopamine	-
Methylxanthine	-	-	Caffeine, paraxanthine
Local anaesthetics		-	Procaine

*Hormones as a class of contaminants in wastewater, warranting specific attention and study

In Table 6, although estradiol was under negative removal, a treatment train involving EQ ➝ AD ➝ MBR ➝ OZ ➝ DGS, had a removal efficiency of 99.9% for the estradiol hormone as shown in Table 3.

## Active pharmaceutical ingredients (*APIs*) in drinking and grondwater

Patterton [69] conducted studies of drinking water from 7 cities in South Africa over 4 seasons and detected various pharmaceuticals in the water as illustrated in Table 7.

**Table 7:** Pharmaceuticals detected in South Africa’s drinking water. Adopted from Swartz et al. [41]

Analyte	Description	Analyte	Description
Benzocaine	Anaesthetic	Phedrin	Bronchodilator
Paracetamol	Analgesic	Diphenylamine	Fungicide
Temazepam	Antianxiety	Imazalil	Fungicide
Flecainide	Antiarrhythmic	Thiabendazole	Fungicide
Nalidixic acid	Antibiotic	Atrazine	Herbicide
Sulfisomidine	Antibiotic	Hexazinone	Herbicide
Carbamazepine	Anticonvulsant/antiepileptic	Metazachlor	Herbicide
Oxcarbazepine	Anticonvulsant/antiepileptic	Metolachlor	Herbicide
Phenytoin	Antiepileptic	Propazine	Herbicide
Fluconazole	Antifungal	Tebuthiuron	Herbicide
Telmisartan	Antihypertensive	Terbumeton	Herbicide
Atenolol	Antihypertensive	Terbuthylazine	Herbicide
Minoxidil	Antihypertensive vasodilator	Imidacloprid	Insecticide
Cinchonidine	Antimalarial	Alachlor	Pesticide
Cinchonine	Antimalarial	Sebuthylazine-desethyl	Pesticide
		Simazine	Pesticide

Swartz et al. [41], highlighted that the ARVs,lamivudine and stavudine, carbamazepine; cinchonidine and cinchonine, paracetamol and sulfamethoxazole (antibiotic) should be prioritised and monitored in potable water for reuse in South Africa. To restrict this list, the authors considered factors such as persistence in the environment, ability to disrupt the endocrine system, widespread use, synthetic or naturally occurring, and whether water-soluble or not. Pharmaceuticals classified as analgesics, anti-inflammatories, and beta blockers were found as the most persistent drugs and had low removal efficiencies in wastewater treatment. According to the Department of Environmental Affairs, other antiretrovirals of significant concern in South Africa are tenofovir, zalcitabine, nevirapine, lopinavir, and didanosine [40].

Bolong et al. [70] reported the growing problem of emerging contaminants and endocrine disruptors including estradiol in Germany, Japan, and the United Kingdom’s drinking water, surface water, and wastewater. Levels of concentration of these substances were dependent on population and consumption behaviour and wastewater treatment performance.

According to Swartz et al. [41], carbamazepine found in South Africa’s drinking water is also a persistent pollutant. In addition, the drug and paracetamol can be used to assess the removal efficiency of pharmaceuticals from wastewater. In countries such as Australia, they are considered in the drinking water quality standards [41]. The Reference Dose given is 0.013 mg/kg/d, based on the dose allowed for children including a measure of uncertainty. In Australia, the guidelines for Water Recycling Augmentation of Drinking water supplies are stricter with a reference dose of 0.0028 mg/kg/d. Patel et al. [16] also tabulated the pharmaceuticals found in various water sources as shown in Table 8. Groundwater which is also used as a source of drinking water by many people is also vulnerable to contamination by APIs and therefore critical to monitor.

**Table 8:** Pharmaceuticals in drinking water and groundwater [16]

Country	Waterbody	Pharmaceutical contaminants	Concentration range (ng/l)
China (Shanghai)	Drinking water	Alprazolam Diazepam Temazepam	2.4 1.9 0.2
China (Beijing)	Drinking water	Bezafibrate Antipyrine Aminopyrine Carbamazepine Ibuprofen	0.31–0.85 0.15–0.22 0.17–0.64 0.37–1.15 < LOD-17.17
China (Beijing)	Tap water	Naproxen Diclofenac Bezafibrate Sulfamethoxazole Carbamazepine Clofibric acid	< LOD-3.12 < LOD-2.37 < LOD-0.16 < LOD-1.81 0.51–38.24 < LOD-1.37
Serbia (Novi Sad, Zrenjanin, Vrbas and Obrenovac)	Drinking water	Ketoprofen Salicyclic acid Carbamazepine 10,11-Epoxycarbamazepine Sotalol Propranolol Metoprolol Hydrochlorothiazide Irbesartan Salbutamol Iopromide Levamisole	16 < LOQ-1.4 < LOQ-8.7 128 0.4 < LOQ-4.3 < LOQ-3,5 24 < LOQ-2.2 < LOQ-5.4 6.8 < LOQ-2.8
Serbia (Novi Sad, Zrenjanin, Vrbas and Obrenovac)	Underground water	Phenazone Propyphenazone Carbamazepine Propranolol Carazolol Albendazole Naproxen Ibuprofen Salicyclic acid	23.4 < LOQ-24.8 3.4 < LOQ-4.5 3.3 1.9 27.6 92 < LOQ-2.5
Spain (Madrid)	Tap water	Caffeine Diatrizoate Iohexol Iomeprol Iopromide	0.47–501.62 0.8–1.1 0.5–5.0 1.1–1.4 0.4–1.0
Taiwan (Taipei and Hsinchu)	Groundwater	Sulfadiazine Sulfamethoxazole Sulfathiazole Sulfamethazine Sulfamonomethoxine Sulfadimethoxine Erythromycin-H_2_O Clarithromycin Nalidixic acid Flumequine Pipemidic acid Norfloxacin Ofloxacin Dimetridazole Metronidazole Atenolol Acebutolol Metoprolol Acetaminophen Ibuprofen Naproxen Diclofenac Clofibric acid Gemfibrozil Bezafibrate Caffeine Carbamazepine Pentoxifylline Lincomycin Trimethoprim Ciprofloxacin Propranolol	0.1–14.4 0.1–1820 0.6–3.0 0.3–28.9 0.6–1.8 0.8–4.3 2.2–54.8 6.9–12.5 1.6–16.4 1.0–317.0 2.6–10.2 2.8–9.3 0.9–11.8 1.8 4.9–35.6 3.6 0.7 7.8 0.9–1036 7.0–836.7 128.0 2.1–33.2 0.1–1.0 0.1–172.3 0.1–256.7 1.2–930.7 0.4–37.9 0.4–2.4 1.1–48.4 0.1–17.8 1.6 5.4
United States of America (New York, Skaneateles Lake)	Tap water	Sulfamethoxazole Triclosan Triclocarban Atenolol Ibuprofen Bisphenol A Oxybenzone Caffeine	ND-0.39 ND-1.93 ND-20.2 ND-19.5 ND-1.16 ND-421 ND-1.42 ND-11.1
Italy (Milan)	Drinking water wells (groundwater)	Caffeine Carbamazepine Clofibric acid	0.31–10.3 1.05 2.4–5.2

* < LOD- Below limit of detection; * < LOQ-Below limit of quantification

With a growing body of evidence that has proven the presence of pharmaceutical contaminants in wastewater, tap water, and groundwater, South Africa is not immune to this problem. However, current conventional treatment techniques involving mechanical, biological, chemical, and physical methods, either individually or in combination, which are being employed, are inadequate in the remediation of these contaminants as various studies have revealed. Most pharmaceutical contaminants and their metabolites which can be more toxic than the original compound, are discharged into water bodies posing an environmental threat [62]. Conventional technologies are also reportedly costly, complex, time-consuming, and require skilled labour to operate [17]. It is therefore critical to evaluate possible technologies that can be employed to improve the removal efficiencies of these contaminants before ingestion by humans and animals and uptake by plants. The availability of monitoring tools or sensors with low detection limits will also be key in detecting these pharmaceutical contaminants.

Table 8 shows that the reported concentration ranges of pharmaceutical contaminants in drinking water and groundwater samples across different countries are generally in the parts per trillion (ng/L) to parts per billion (μg/L) level. While these levels may appear low, they are still concerning from an environmental and public health perspective. Comparing the reported values to WHO drinking water guidelines, where available, reveals the following:

- Carbamazepine levels in drinking water samples from China and Serbia exceed the WHO provisional guideline value of 1 μg/L.
- Sulfamethoxazole levels in groundwater from Taiwan and the USA (New York) are within the range of the WHO provisional guideline value of 0.5–10 μg/L.
- For most other pharmaceuticals, there are no specific WHO guideline values available, as the potential health effects at these low concentrations are still being researched.

This indicates that the presence of pharmaceutical contaminants in drinking water and groundwater is a significant challenge that requires further investigation and implementation of advanced treatment technologies. The low-level concentrations, while potentially not acutely harmful, can still pose long-term risks through chronic exposure, particularly for vulnerable populations. Continuous monitoring and development of effective removal methods are crucial to ensure the safety of water resources.

## The emergence of green technologies in the removal of APis

Green technologies involving physical, biological, and chemical processes have been touted as potential treatment technologies in reducing organic and inorganic pollutants in water and wastewater [71]. Sustainable treatment methods aim to minimise operational costs while simultaneously reducing residual waste volume and its toxicity profile [72]. The treatment method selected depends on factors such as wastewater composition, process efficiency, capital, and operational costs, availability of the technology, complexity or simplicity of the plant, and the environmental impact of the process. However, it should be noted that treatment processes are coupled in most cases to improve removal efficiencies [72]. Examples of such green technologies include the use of enzymes, sunlight, electrons, microorganisms, and solid waste residue from industrial, agricultural, or municipal processes. Some of these processes are briefly discussed in the following section.

### Nanotechnology

The introduction of nanotechnology in wastewater treatment has also improved the sustainability and efficiency of existing treatment processes through the manufacture of membranes, adsorbents, nano-catalysts, nanostructured surfaces, and reagents [72, 73]. The interest in nanoadsorbents has been increasing due to the reported effectiveness in removing pharmaceutical contaminants compared to conventional adsorbents, and their relatively low cost of manufacture. Nanoadsorbents have a higher specific surface area, are highly substance selective, and may have various combined reactive agents [73, 74]. Khan et al. [73] reviewed various green nanoadsorbents that can be used to treat pharmaceutical-contaminated water and wastewater. The authors cite various researchers who assessed the removal of these contaminants using nanoadsorbents synthesised from haematite, magnetite, Tilia leaves (copper nanoparticles), sodium silicate (nanosilica), wood, coal, or plastic (activated carbon), anionic clay and its nanocomposites and biochar-based sorbents. Carbon nanotubes and zeolites (natural and synthetic) are among the adsorbents of interest in treating pharmaceutical-contaminated waters [17]. Cincinelli et al. [17] also highlight the importance of preventing the discharge of nanomaterials into the environment when used in water treatment. This can be done by enveloping the nanoadsorbent in a particle of larger diameter or placing the adsorbent on supporting substrates [17].

Due to their low molecular weight cut-off, nanofiltration membranes have also shown promise in removing these contaminants [75]. Furthermore, charged molecules can be removed through the interaction of electrostatic forces, while uncharged molecules are adsorbed onto the surface [75–77]. As nanomembranes are susceptible to fouling [75], it is vital also to study the optimal conditions that minimise fouling without compromising the membrane's selectivity.

- Nanoadsorbents made from materials like haematite, magnetite, Tilia leaves (copper nanoparticles), sodium silicate (nanosilica), wood/coal/plastic (activated carbon), anionic clay, and biochar have demonstrated effective removal of pharmaceutical contaminants from water and wastewater compared to conventional adsorbents.
- Carbon nanotubes and natural/synthetic zeolites have also shown promise in treating pharmaceutical-contaminated waters.
- Nanofiltration membranes with low molecular weight cut-offs can remove these contaminants through size exclusion and electrostatic interactions.
- To prevent the discharge of nanomaterials, they can be enveloped in larger particles or immobilized on supporting substrates.

### Microalgal treatment

Microalgae can remove contaminants through bioadsorption, bioaccumulation and biodegradation, with biodegradation being the most common. Removal efficiencies of around 80% have been reported for biodegradation. The advantages of microalgal treatment include the potential to utilise solar energy, low cost and environmental benefit. De Salles Pupo et al. [62] cite Hom-Diaz et al. [78], who assessed the removal efficiency of pharmaceutical contaminants from wastewater using a tubular microalgae reactor. Efficiencies varied from 98%, 48%, and 30–57% for anti-inflammatory drugs, antibiotics, and antipsychotic drugs, respectively. Pharmaceutical compounds bearing cationic groups can undergo removal using bioadsorption through the interaction of electrostatic forces. Microalgal treatment can be combined with advanced oxidation processes, microbial fuel cells, and wetlands [62].

- Microalgae can remove pharmaceutical contaminants through biosorption, bioaccumulation, and biodegradation, with reported removal efficiencies up to 98% for some drugs.
- Advantages include utilization of solar energy, low cost, and environmental benefits.
- Microalgal treatment can be combined with other green technologies like advanced oxidation, microbial fuel cells, and constructed wetlands.

### Reverse osmosis

With an even smaller molecular weight cut-off than ultrafiltration, microfiltration and nanofiltration membranes, reverse osmosis has also been recognised as a potential membrane treatment method for removing pharmaceutical contaminants [76, 77]. Reverse osmosis (RO) is a highly effective membrane-based treatment technology that has been recognized for its ability to remove a wide range of pharmaceutical contaminants from wastewater. Compared to other membrane filtration methods, such as ultrafiltration, microfiltration, and nanofiltration, reverse osmosis has an even smaller molecular weight cut-off, allowing it to effectively remove a broader range of pharmaceutical compounds [79].

The high removal efficiency of reverse osmosis is due to its ability to reject a variety of contaminants based on their size, charge, and other physicochemical properties. Studies have shown that reverse osmosis can achieve removal rates of up to 99% for various active pharmaceutical ingredients (APIs), including antibiotics, antidepressants, hormones, and other emerging contaminants [80]. The performance of reverse osmosis in removing pharmaceutical contaminants is influenced by several factors, such as the membrane characteristics, operating conditions (e.g., pressure, temperature, and flow rate), and the properties of the pharmaceutical compounds (e.g., molecular weight, charge, and hydrophobicity). Optimization of these parameters can further enhance the removal efficiency of reverse osmosis for specific pharmaceutical contaminants [79]. In addition to its high removal efficiency, reverse osmosis also offers the advantage of producing a high-quality permeate, which can be suitable for various water reuse applications, such as irrigation, industrial processes, or even potable water production, depending on the level of post-treatment required [80]. The use of reverse osmosis has been recognized as a promising technology for the treatment of pharmaceutical industry wastewater, effectively removing a wide range of pharmaceutical contaminants and contributing to the overall goal of sustainable water management in the pharmaceutical sector.

## Conclusion

The presence of active pharmaceutical ingredients (APIs) in drinking water sources has become a growing concern worldwide, with numerous studies confirming their ubiquitous occurrence. While the potential health and environmental impacts of long-term exposure to these contaminants are still being investigated, it is clear that effective treatment solutions are necessary to mitigate this issue. The application of green technologies, such as nanotechnology, membrane filtration, and microalgal treatment, has shown promising results in removing a wide range of pharmaceutical compounds from water and wastewater. Nanoadsorbents, nanofiltration, and reverse osmosis membranes have demonstrated high removal efficiencies, while microalgal systems leverage natural metabolic pathways for biodegradation and biosorption. These innovative approaches offer sustainable alternatives to conventional treatment methods, which often struggle to completely eliminate APIs. Moving forward, a comprehensive and multidisciplinary approach is required to address the challenge of API contaminants in drinking water. Governments and regulatory bodies should prioritize the inclusion of APIs in routine water quality monitoring programs, establish stricter guidelines and limits, and ensure strict compliance. This will provide the necessary data and impetus to drive further research and technology development.

Future research should focus on the following key areas:

- Comprehensive monitoring and risk assessment: Expanding the scope of water quality monitoring to include a wider range of APIs, evaluating their fate and transport in the environment, and assessing the potential risks to human health and aquatic ecosystems.
- Optimization and scale-up of green technologies: Continuous improvements in the design, performance, and cost-effectiveness of nanoadsorbents, membrane filtration systems, and microalgal treatment processes to facilitate large-scale implementation.
- Synergistic treatment approaches: Exploring the integration of green technologies with other advanced oxidation processes, bioremediation methods, and hybrid systems to enhance the overall removal efficiency of APIs.
- Environmental fate and impact studies: Investigating the long-term fate and effects of APIs and their transformation products in the environment, including potential bioaccumulation, ecosystem disruption, and development of antimicrobial resistance.
- Policy and regulatory frameworks: Establishing comprehensive regulatory guidelines and enforcement mechanisms to mandate the inclusion of APIs in water quality standards and drive the adoption of advanced treatment technologies.

## Data Availability

No data were generated or analyzed for this conceptual/review article and no ethical clearance was required for the study.
